# Influence of Nitrogen and Phosphorus on Microalgal Growth, Biomass, Lipid, and Fatty Acid Production: An Overview

**DOI:** 10.3390/cells10020393

**Published:** 2021-02-14

**Authors:** Maizatul Azrina Yaakob, Radin Maya Saphira Radin Mohamed, Adel Al-Gheethi, Ravishankar Aswathnarayana Gokare, Ranga Rao Ambati

**Affiliations:** 1 Institute for Integrated Engineering, Universiti Tun Hussein Onn Malaysia, Parit Raja, Batu Pahat 86400, Johor, Malaysia; maizatulazrina@gmail.com; 2 Micropollutant Research Centre (MPRC), Faculty of Civil Engineering and Built Environment, Universiti Tun Hussein Onn Malaysia, Parit Raja, Batu Pahat 86400, Johor, Malaysia; adelalghithi@gmail.com; 3 C. D. Sagar Centre for Life Sciences, Dayananda Sagar College of Engineering, Dayananda Sagar Institutions, Kumaraswamy Layout, Bangalore 560078, Karnataka, India; rgokare@yahoo.co.in; 4 Department of Biotechnology, Vignan’s Foundation of Science, Technology and Research (Deemed to be University), Vadlamudi 522213, Guntur, Andhra Pradesh, India

**Keywords:** microalgae, nitrogen, phosphorus, biomass, lipids, fatty acids

## Abstract

Microalgae can be used as a source of alternative food, animal feed, biofuel, fertilizer, cosmetics, nutraceuticals and for pharmaceutical purposes. The extraction of organic constituents from microalgae cultivated in the different nutrient compositions is influenced by microalgal growth rates, biomass yield and nutritional content in terms of lipid and fatty acid production. In this context, nutrient composition plays an important role in microalgae cultivation, and depletion and excessive sources of this nutrient might affect the quality of biomass. Investigation on the role of nitrogen and phosphorus, which are crucial for the growth of algae, has been addressed. However, there are challenges for enhancing nutrient utilization efficiently for large scale microalgae cultivation. Hence, this study aims to highlight the level of nitrogen and phosphorus required for microalgae cultivation and focuses on the benefits of nitrogen and phosphorus for increasing biomass productivity of microalgae for improved lipid and fatty acid quantities. Furthermore, the suitable extraction methods that can be used to utilize lipid and fatty acids from microalgae for biofuel have also been reviewed.

## 1. Introduction

The size of microalgae generally ranges between 1 µm and 2 mm. They can utilize light and carbon dioxide through the photosynthetic process to multiply and produce biomass [1,2]. Generally, microalgae are unicellular aquatic microorganisms that can be cultivated in a wide range of environmental conditions, either in saltwater, freshwater or wastewater, due to their high tolerance to environmental stress [3,4]. Different cultivation conditions of microalgae may affect microalgal growth rates, biomass yield and their nutritional content in terms of lipid and fatty acid production [3,5,6,7,8].

Microalgae are adopted to reduce pollution in waterways by a phycoremediation process to purify contaminants from water bodies and wastewater. Jais et al. [9] and Yaakob et al. [10] have reported microalgae-mediated reduction in pollutant levels in water bodies by the degradation of organic matters, uptake of heavy metals and regulation of chemical oxygen demand (COD) and biological oxygen demand (BOD) to the levels which are safe to be discharged. *Scenedesmus* sp. was adapted to treat swine wastewater wherein the total phosphorus (TP), total nitrogen (TN) and total carbon (TC) in swine wastewater were reduced up to 83%, 87% and 12%, respectively, for 40 days through the phycoremediation process [11,12].

Microalgae generally comprise 28–70% of protein (on a dry matter basis), 10–20% of lipids and 10–50% of carbohydrates, respectively [13,14,15]. Studies by Hakalin et al. [16] and Baharuddin et al. [17] demonstrated that microalgae can be used in aquaculture for fish and shrimp feed due to their size, and are easily consumed. Microalgal feeds also promote rapid growth rates and high nutritional content in terms of lipids, proteins, carbohydrates, amino acids and fatty acids in the aquaculture-cultivated fish and shrimps.

Microalgae can be used to produce biofuel as an alternative energy source due to their high lipid content in the cells with an additional advantage of minimal land usage compared to terrestrial plants [4,18]. Few studies have demonstrated that microalgae can produce 58,700 L/ha of oil for biofuel, which is relatively higher compared to other crops such as jatropha and canola, with 1892 L/ha and 1190 L/ha, respectively [18,19]. High lipid content in microalgal biomass is a prime factor of its suitability to produce biofuel. Zhang and Hong [20] reported that *Scenedesmus* sp. yielded 22% lipids from their biomass. The main factors which may influence microalgae cultivation are nutrient content such as nitrogen and phosphorus, followed by mode of cultivation, temperature, light intensity, light:dark period, salinity, pH, mixing, turbulence and also carbon dioxide concentration [7,21]. Nitrogen and phosphorus are essential macronutrients needed to promote algal growth and they regulate metabolic activities if supplied in an acceptable form. Various nitrogen and phosphorus concentrations in microalgae cultivation medium may influence lipid and fatty acid yield [4,18]. According to Munoz and Guieysse [22], microalgae can take up high nitrogen and phosphorus concentrations for the buildup of protein and nucleic acid synthesis in up to 40–60% of their dry weight.

This overview deals with the technical feasibility of utilization of nitrogen and phosphorus for microalgae growth, biomass production and their influence on nutritional composition, including lipid and fatty acids. The main aim of this review is to provide a summary of recent information concerning the role of nitrogen and phosphorus in microalgae cultivation that influences the growth of the organism, biomass yield, and productivity of lipid and fatty acids.

## 2. Microalgae Nutrient Composition

Several factors may influence microalgal growth, biomass, and lipid and fatty acids production. However, this review paper will focus only on how the limitation of nutrients (nitrogen and phosphorus) affects microalgal growth and its biochemical composition as studied in different microalgae species [4,5,6,7,23].

### 2.1. Role of Nitrogen

Nitrogen is an essential macronutrient for microalgal growth and plays an important role in protein, lipid and carbohydrate synthesis [5,7]. Generally, nitrogen concentration significantly influences microalgal growth and their biochemical compositions; at the same time, depletion of nitrogen in cultivation medium causes a decrease in growth with concomitant increases in d lipid productivities [24]. Microalgae can assimilate nitrogen in the form of nitrate, nitrite, urea and ammonium [25]; nevertheless, nitrate is widely used for microalgae culture compared to ammonium salts as it is more stable with less likelihood of pH shift. Moreover, ammonia (NH_4_^+^) concentrations greater than 25 μM are toxic for microalgae; thus, nitrate (NO_3_^−^) is used commonly in culture media [26]. However, the limitation of nitrogen in the culture medium may lower biomass production but would enhance lipid production. This was demonstrated by Xu et al. [25] who observed that a drop in pH in ammonium-supplemented cultivation medium of *Ellipsoidion* sp. caused a reduction in growth rate with simultaneous enhancement in eicosapentaenoic acid (EPA) and polyunsaturated fatty acids (PUFA) accumulation. Yang et al. [4] showed that biomass accumulation in *Chlamydomonas reinhardtii* was inhibited under nitrogen deficiency up to 31.7%, with simultaneous increases in total fatty acid yield up to 93%, coupled to enhancement in lipid production up to 113.46 ± 1.78 mg/L. Thus, it can be concluded that nitrogen concentration favors higher biomass productivity, and depletion of nitrogen shifts the flux to lipid production [4,5,7,8]. Table 1 shows microalgae cultivation in correlation with nitrogen content.

Zhu et al. [3] reported that *Chlorella zofingiensis* showed rapid growth in nitrogen-sufficient culture medium, whereas growth inhibition under nitrogen starvation conditions was observed. Upon nitrogen starvation, lipid accumulation increased greatly in *C. zonfingiensis* cells, which is desirable for biodiesel generation. The results obtained by Zhu et al. [3] were similar to that of Zarrinmehr et al. [7] who reported that the cell cultures of *Isochrysis galbana* showed reduced growth in nitrogen-deficient conditions; and in contrast, carbohydrates and fatty acids levels enhanced by 47% and 75%, respectively. Microalgae growth in nitrogen-deficient medium might change their lipid metabolic pathway and accumulate mainly triacylglycerides (TAGs), which are stored in the cytoplasm of microalgae as a source of carbon and energy [4,28]. Nitrogen-limiting conditions can decrease the thylakoid membrane cellular content, activates acyl hydrolase and stimulates phospholipid hydrolysis, resulting in increased intracellular content of fatty acyl-CoA [28].

Generally, nitrogen starvation is an effective technique to be used to increase lipid production in microalgae cells. Nitrogen-deficient culture conditions produced two-fold lipids compared to nitrogen-sufficient medium as reported by Jia et al. [30] and Pancha et al. [29]. However, nitrogen depletion conditions can lead to the reduction of microalgae biomass productivity [33]. *I. galbana* was cultivated in different nitrogen concentrations to induced stress for lipid production. The result reported that the lipid production in *I. galbana* decreased when nitrogen sources were below 288 mg/L. According to Zarrinmehr et al. [7], the highest and lowest nitrogen concentration in culture medium triggered stress to microalgae cells. On the other hand, increased nitrogen concentration from 72 mg/L to 144 mg/L may enhance biomass production in *I. galbana*. This observation is similar to that of Feng et al. [34], who reported that *C. zofingiensis* was able to generate 65% lipids under nitrogen-deficient conditions. Table 2 summarizes the effect of nitrogen availability and starvation conditions on biomass yield, lipid content and fatty acids in algae.

Based on Table 2, it is clear that nitrogen plays an important role in influencing the fatty acids profile and the trends for fatty acids production for nitrogen starvation, and nitrogen availability conditions are quite similar with the highest yield of C14:0, C16:0 and C18:0 [3,5,8,35,36,37,38,39,40]. This statement was supported by Savvidou et al. [41], who reported that nitrogen limitation conditions will generate higher saturated fatty acids such as C14:0, C16:0 and C18:0. Delgado et al. [40] reported that at 9.375 mg/L nitrogen content, *Picocystis salinarum* produces 72.13 µg/g, 923.95 µg/g and 19.87 µg/g of C14:0, C16:0 and C18:2 fatty acids, respectively.

### 2.2. Role of Phosphorus

Phosphorus is another essential compound that plays a significant role in algal growth, lipid production, fatty acid yield and metabolic processes such as energy transfer, signal transduction and photosynthesis [6,42]. Phosphorous is an essential nutrient that makes up slightly less than 1% of total algal biomass and is required at approximately 0.03–0.06% in the medium to sustain algae growth [26,42,43]. Phosphorous is essential to microalgal cells for the production of cellular components such as phospholipids, DNA, RNA and ATP for the metabolic pathways that involve energy transfer and nucleic acid synthesis [44]. Microalgae can uptake phosphorus in the form of polyphosphate or orthophosphate to enhance their growth and nutritional content in the cells.

Polyphosphate in microalgae represents another form of cell protection from metal toxicity as it can bind with incoming metals into a detoxified complex [26]. *C. reinhardtii* accumulated copper (Cu) and cadmium (Cd) and survived the metals’ toxic effects in polyphosphate-rich conditions [26]. However, some microalgae could accumulate more phosphorus in the form of orthophosphate (PO_4_^3−^) and polyphosphate than required for growth as they can be converted to ATP under unfavorable nutritional conditions. The ATP is needed for pumping of the polyphosphate into the microalgae cell and for the conversion of phosphorus to polyphosphate. The source of energy for ATP is the photosynthetic process and also respiration [2,28,45]. Table 3 shows microalgae cultivation related to various phosphorus content in the medium.

Mulbry et al. [52] showed that microalgae have high efficiency to absorb inorganic phosphate from wastewater by luxurious uptake in the range of 70% to 90% compared to optimal levels needed for their growth [53]. It is also reported that microalgae growth and phosphate uptake are linearly proportional to the biomass yield. Chu et al. [28] demonstrated that *Chlorella vulgaris* had been cultivated for biodiesel production under phosphorus-sufficient conditions. The result showed that the lipid yield of *C. vulgaris* for biodiesel production was 58.39 mg/L/day in phosphate-sufficient conditions, which was relatively higher than in a phosphate-deficient regime. Thus, it can be concluded that phosphate is an important macronutrient for microalgal lipid production [28]. However, the phosphorus uptake by microalgae can reach saturation due to the limitation of light, and reduction of carbon dioxide and oxygen levels in the culture medium.

Phosphorus is an essential nutrient for microalgae growth and cell division, and its requirement is varied in different microalgae species. According to Roopnarain et al. [47] the optimum phosphorus concentration for microalgae is in the range of 0.001 g/L to 0.179 g/L. Phosphorus limitation is an efficient environmental pressure to induce lipid accumulation [6,18]. The lipid content in *Scenedesmus* sp. has increased under reduced phosphorus levels in the medium. The *Scenedesmus* sp. grown in 50 mg/L phosphorus achieved 22.3% lipid content, whereas lipid yield reached 42.5% in 1 mg/L phosphorus [6]. Similarly, *Rhodosporidium kratochvilovae* under limited phosphorus concentration (0.05 g/L) produced 51.7 ± 0.81% lipid content after eight days of cultivation [32]. Under the limitation of phosphorus, *R. kratochvilovae* formed big irregular-sized lipid droplets (LD) and enhanced TAG accumulation, which improves the biodiesel production properties like oxidative stability, viscosity, cetane number and cold filter plugging point [32].

Phosphorus-limited conditions in *I. galbana* showed that the phosphorus starvation medium (0%) and limited P medium (12.5%) induced high lipid content up to 50% (*w*/*w*) on 14 days of cultivation [47]. Besides that, the study also reveals that under stress conditions, *I. galbana* tends to use all available phosphorus in their cell for survival. Limited phosphorus concentrations could support continuous cell growth as well as enhance lipid accumulation in microalgae by switching photosynthetic carbon partitioning toward energy-rich storage macromolecules, mainly lipids, under stress conditions. Carbon partitioning blocks CO_2_ and starch synthesis, driving the metabolism towards lipid production for energy storage in phosphorus stress conditions [6,18]. The observation was corroborated by Feng et al. [34], who showed that phosphorus-limited conditions were suitable for lipid generation in *C. zofingiensis*. Table 4 summarizes the effect of nitrogen availability and starvation conditions on biomass yield, lipid content and fatty acids in algae.

It is evident from Table 4 that the fatty acids profile in microalgae cultivated in phosphorus-replete and -deplete conditions are predominantly of C16:1 and C18:1 [4,6,32,41,49,54,55,56,57].

## 3. Microalgae Cultivation Medium

Microalgae commercialization is limited due to high operational costs such as labor, materials and chemicals, facilities, energy, low biomass yields and also the contamination risks [58,59]. Therefore, it is necessary to have a cultivation system that is efficient to reduce production costs by enhancing biomass productivity. Conventionally, microalgae were cultivated in open ponds and also tubes for biomass production [60,61]. However, this conventional cultivation technique has several drawbacks such as the risk of contamination by bacteria and protozoa and costs of electricity and water [62]. To encounter some of these drawbacks, the algae biomass production may be undertaken using wastewater medium [63], coupled to low energy-intensive harvesting methods and also effective extraction procedures [64].

Microalgae cultivation in large scales consume less land compared to the other crops, and the biofuels obtained from microalgae biomass are known as carbon neutral fuels as they use atmospheric carbon during photosynthesis [65]. Singh et al. [37] demonstrated that the lipid productivity of *Ankistrodesmus falcatus* had been influenced by nutrient-rich cultivation medium consisting of nitrogen and iron such as nitrogen 750 mg/L and iron (Fe) 9 mg/L, wherein fatty acid production was 59.6%, 74.07 mg/L/d and 38.49%, for N, P and Fe, respectively. Similarly, Ji et al. [66] studied the effect of the starvation of nitrogen, phosphorus and sulfur in the cultivation medium on growth and lipid production in *Tetrasemis* *subcordiformis*. The results revealed that the cell growth in *T. subcordiformis* slowly increased during nutrient deprivation, indicating that the nutrients reservoir inside its cells facilitates sustenance of growth until the availability of the essential nutrients [66]. Interestingly, higher nitrogen (N) to phosphorus (P) ratio in the culture medium caused phosphorus deprivation for microalgae growth [65].

### 3.1. Growth Rates

The composition of nitrogen and phosphorus relatively affect microalgae growth [18,26,67]. According to Zhu, [3] growth of *C. zofingiensis* was severely inhibited upon nitrogen depletion and the results were similar to a study carried out by Ji et al. [66] in *Tetrasemis subcordiformis.* Besides that, low growth rates in nitrogen-deficient conditions in microalgae tend to accumulate higher saturated fatty acids (SFA) and monounsaturated fatty acids (MUFA) into neutral lipids. Whereas, higher growth results in a high accumulation of polyunsaturated fatty acids (PUFA) [68].

**Figure 1:**
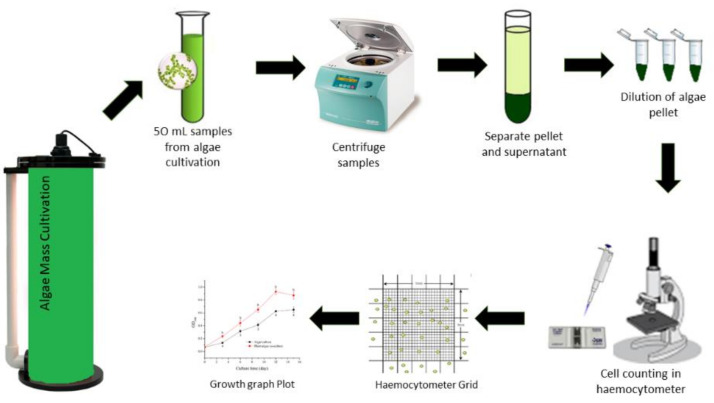
Microalgae cell counting for growth rate determination.

Here we discuss the measurement of growth of algae for determining the biomass productivities. Firstly, the growth of microalgae can be determined by cell number counting using a hemocytometer after appropriate dilution (Figure 1).

Based on Figure 1, the microalgae cell concentration was calculated using Equation (1), where *D_f_* was the dilution factor of microalgae. Microalgae growth rates were measured based on maximum growth rate (*μ_max_*), division per day (*D_d_*) and doubling time (*t_d_*) according to the Equations (2), (3) and (4), respectively, where *X_m_* is a maximum cell number, *X_0_* is an initial cell number, *t_m_* is the day of maximum cell number and *t_0_* is day of initial cell number.

Concentration algae (cell/mL) = 𝐴𝑣𝑒𝑟𝑎𝑔𝑒 𝑛𝑜 𝑜𝑓 𝑐𝑒𝑙𝑙 ×𝐷_𝑓_ × 10^4^ (1)

Maximum growth rates (*µ_max_*) = (In (*X_m_* − *X_0_*))/(*t_m_* − *t_0_*) (2)

Division per day (*D_d_*) = *µ_max_*/In2 (3)

Doubling time (*t_d_*) = 1/*D_d_* (4)

Anand and Arumugam [36] studied *Scenedesmus quadricauda* growth in Bold’s basal medium (BBM) medium rich in nitrogen and in nitrogen-depleted medium to evaluate the effect of nitrogen on cell growth and cell density. The cell number was determined for 30 days and at every three (3)-day interval, using hemocytometer microscopic cell count. The study result reported that *S. quadricauda-*specific growth rates and doubling time were higher in nitrogen-rich conditions (0.33µ/d, 2.09/d) compared to nitrogen-depleted conditions (0.14 µ/d, 4.93/d). Thus, the reports indicate that microalgae growth density was directly proportional in the presence of nitrogen [3,35,40] and nutrient manipulation is the most efficient tool for the modulation of microalgae growth and biomass composition [26].

### 3.2. Biomass Quality

**Figure 2:**
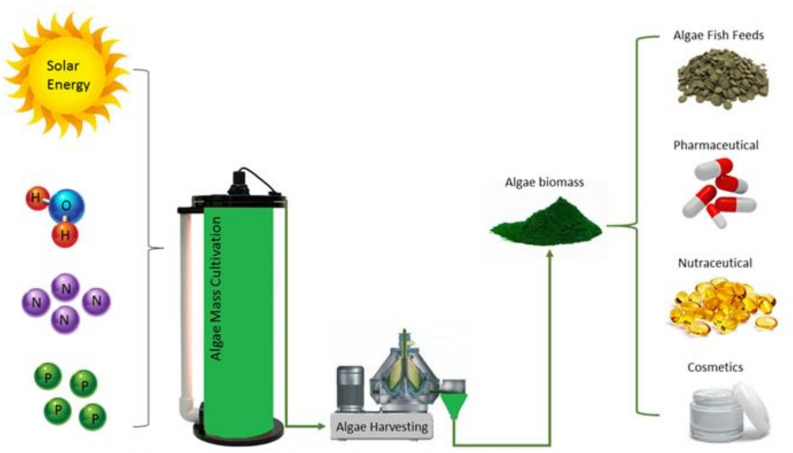
Microalgae cultivation with availability of essential nutrients and potential bioproducts.

Microalgae are a promising source of food, biofuel, animal feed, fertilizer, cosmetics and also pharmaceuticals (Figure 2). The quality of these bioproducts depends on the quality of microalgae biomass composition. Microalgae biomass can be quantified by using several techniques such as cell counting, determination of volume, optical density and weight [64,69]. Generally, microalgae biomass and its quality are closely related to microalgae growth rates. A higher growth rate would provide higher microalgal biomass in a short period of time.

**Figure 3:**
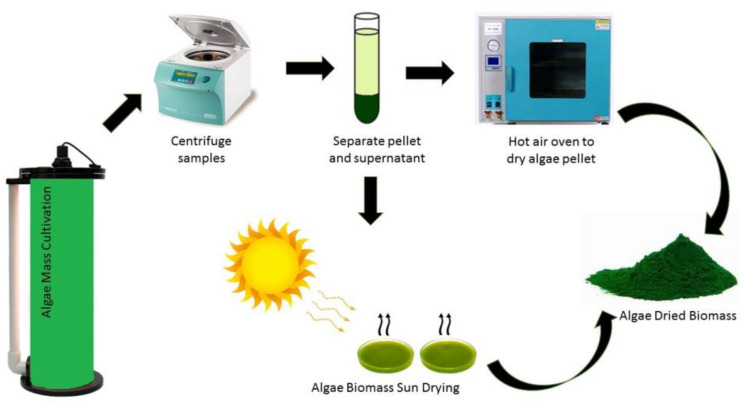
Microalgae harvesting and biomass production.

To measure microalgae biomass, microalgae culture may be filtered through a preweighed Whatman GF/C filter paper and then the filter paper is dried in an oven [3,6,18,24]. The difference between the final weight and weight before filtration is the dry biomass weight of the microalgae. Besides that, microalgae biomass also can be determined by sun drying as illustrated in Figure 3. However, the sun drying method is not effective in high humidity countries due to the high water content in microalgae biomass, often leading to unpleasant odors due to potential bacterial contamination [61].

Microalgae biomass productivity is dividing the biomass with number of cells concentration in culture. Microalgae biomass productivity can be determined according to kinetic parameter Equation (5), where *µ_max_* is the maximum microalgae growth rate, *X_0_* is initial cell concentration and *X_m_* is maximum cell concentration in culture. The productivity was recorded in cells per milliliter per day (cells/mL/day).

Biomass productivity = (*µ_max_* (0.9*X_m_* − 1.1X*_0_*))/(In (9(*X_m_* − 1.1X*_0_*)/(1.1X*_0_*))) (5)

Generally, the productivity of biomass and lipid is influenced by nutrients and environmental factors [70]. In normal cultivation conditions, starch is the primary energy storage form in the photosynthetic fixation of carbon dioxide [71,72]. Starch stored in chloroplasts provides energy for vital metabolic processes, besides functioning as a carbon storage sink [71].

In nutrient depletion conditions, the microalgae growth and cellular division were inhibited, diverting the storage carbon to metabolic processes away from growth [71,72]. Starch accumulation can be an indicator of the balance between material and energy supply during the photosynthesis process. On the other hand, starch production decreased dramatically during nuclear and cellular divisions (growth process) as indicated in cytological studies [71]. This condition was able to divert stored carbon to lipid metabolism and to produce a range of secondary compounds. Lipids are another means of energy and carbon storage [72]. The shift from primary storage to secondary storage was due to lipid characteristics that are mainly made up of saturated and monounsaturated fatty acids which can be packed into the cell and generate more energy via oxidation than starch [71,73].

The decrease in the content of chlorophyll pigment is known as chlorosis and it is associated with nutrient depletion. Chlorophylls give microalgae a bright green color [71] and the diversion of microalgae starch production to lipid accumulation is initiated by carbon metabolism flux, also known as the oleaginicity process. The microalgae cells stopped growth and did not divide once the cultivation medium was completely depleted in nutrients, even though they had sufficient energy reserves to perform reproduction (cell division) [72]. Studies by Fernandes et al. [73] reported that starch content in *Parachlorella kessleri* decreased from 25% to 10% of dry weight (DW), while lipids increased from 0% to 29% of DW. Oleaginicity of central carbon metabolism could influence carbon partitioning towards TAG synthesis and thus reduce microalgae growth rate because TAG synthesis requires a carbon supply that supports microalgae growth, such as acetyl-CoA and glycerol 3-phosphate (G3P) [74]. G3P is derived from dihydroxyacetone phosphate (DHAP) produced from the Calvin cycle and glycolysis. Whereas, acetyl-CoA is a central metabolite for biochemical reactions, linked with anabolism and catabolism of TAG synthesis and the tricarboxylic acid (TCA) cycle [70,74].

TAG synthesis is an efficient way to protect against the production of reactive oxygen, photodamage of microalgal cells and protection against stressful conditions inducing nutrient deficiency conditions [68]. TAG was overproduced when their growth was retarded due to nutrient limitation but still had sufficient energy and carbon for metabolism. Generally, this condition triggered higher lipid production, leading to higher saturation of fatty acid [68].

It is ideal to achieve high microalgae productivity (biomass) and a high content of carbon and energy storage in terms of starch and lipids for industrial application [72]. Moreover, the algal biomass could be used for the production of bioenergy such as biogas, biofuel and biomethane, which can be an alternative renewable energy source instead of fossil fuels.

## 4. Microalgae Lipids and Fatty Acids Extraction Methods

The lipid extraction method is an important process for the production of microalgae biofuels and there are several techniques used to determine lipids and fatty acids concentration from microalgae biomass such as supercritical fluids, pressurized solvent extraction, infrared spectroscopy and gas chromatography-mass spectrometry (GC-MS). This subsection will critically discuss analytical techniques previously used by researchers to extract lipid and fatty acids.

### 4.1. Lipids Extraction Methods

Lipids are essential compounds in the microalgae biomass as they comprise triacylglycerols (TAG), phospholipids and glycolipids [2,5,68], which are essential for metabolic activities and biofuel production. Usually, microalgae were associated with high costs for biomass downstream processing for oil extraction. Thus, it is important to extract lipids from microalgae cells using suitable technology that is cost-effective and environmentally friendly. Several methods were applied to quantify lipids from microalgae such as near infrared spectroscopy at specific wavelengths, electroporation, the supercritical fluid extraction method (SEF), pressurized solvent extraction, organic solvent and also osmotic shock [2,64,75] as in Table 5.

Kumar et al. [64] stated that electroporation, pressurized solvent extraction and the supercritical fluid extraction method are the most efficient technologies to extract lipids from microalgae biomass. However, they required high energy with high operational costs. Generally, cost-effective methods are used for lipid extraction such as osmotic shock, organic solvents through Soxhlet extraction, Bligh–Dyer method and Folch method [18,24,75].

**Figure 4:**
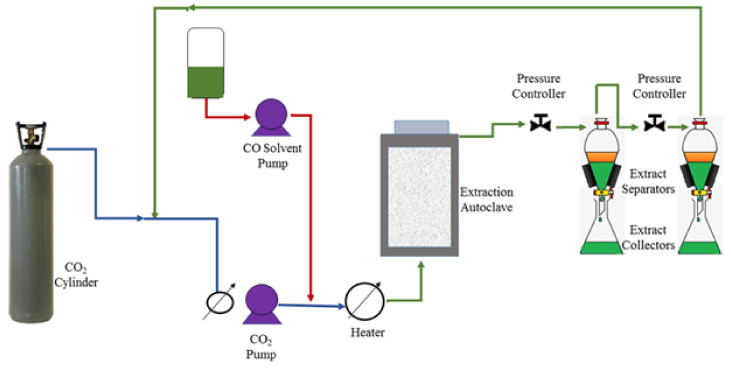
Microalgae supercritical fluid extraction method.

According to King, [76] the supercritical fluid extraction method (SEF) can be used to extract lipids without using any organic solvent for analysis. This SEF method requires high energy consumption that is expensive for commercialization. The SEF method can separate lipids according to properties, enrichment, hydrogenation and hydrolysis. Figure 4 illustrates microalgae lipid extraction using SEF methods. Santana et al. [77] reported the use of the SEF method to extract lipids from *Botryococcus braunii* for biodiesel production. They observed that the lipid yield decreased with an increase in the temperature of extraction. However, the pressure enhancement has a positive influence on lipids productivity. The optimum operating conditions for SEF was in the range of 220–250 bar for pressure and 50 °C for temperature, respectively. Similar results were carried out in *Nannochloropsis salina, Scenedesmus obliquus and Scenedesmus obtusiusulus* for extraction of lipids to generate biodiesel [52,78]. SEF optimum conditions to generate 92% of lipids was performed at 12 MPa, 20 °C, and a CO_2_ to biomass ratio of 100 [52].

**Figure 5:**
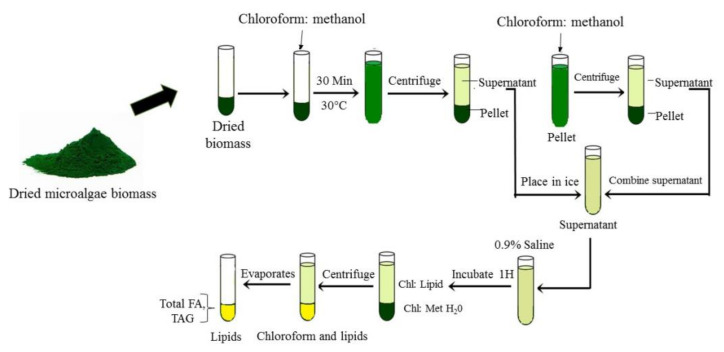
Microalgae lipid extraction by Bligh and Dyer and Folch methods.

Other than SEF, the most common methods used for lipid extraction are modified Folch and Bligh and Dyer methods as shown in Figure 5. The harvested microalgae biomass was added with methanol and chloroform (2:1 v/v) for lipid extraction. Then, lipid extracts were washed with 0.9% saline followed by vortex and separation phase either using thin layer chromatography (TLC) or chromatography (GC-MS). Zienkiewicz et al. [8] followed this method for *Nannochloropsis oceanica:* total lipid quantification and after centrifugation separation, lipids were visualized by thin layer chromatography (TLC). Similarly, Vooren et al. [24] had measured lipid content in *Nannochloropsis oculata* using the Bligh and Dyer extraction method followed by high performance thin layer chromatography (HP-TLC). Vooren et al. [24] showed that *C. zofingiensis* cultivated under nitrogen starvation conditions produced triglycerides in the range of 31% to 43% due to a shift in cell metabolism. Zhu et al. [3] used GC-MS to analyze lipid content in *C. zofingiensis*. The Folch et al. [79] and Bligh and Dyer [80] methods were used for total lipid recovery and extraction was used; it was widely performed by researchers due to the simple procedures, time efficiency, energy savings and suitability for a large sample size.

Another promising method for lipid extraction was an osmotic shock that degrades microalgae cell structures to increase lipid yield efficiency. Gonzalez-Gonzaález et al. [81] studied the effect of osmotic shock on *Dunaliella salina* and *Chaetoceros muelleri* for biogas (methane) generation. The study reported that *C. muelleri* lipid recovery efficiency for methane production was higher than *D. salina* with 72% and 21%, respectively. Microalgae biomass can be used for biomethane generation through anaerobic digestion or the biomass gasification and methanation process. Biomass produced in limited nitrogen culture media was more stable with low inhibitory substances such as ammonia which caused acidosis. Microalgae with high lipids content will yield greater volumes of biomethane than microalgae rich in carbohydrates and protein [82]. Klassen et al. [83] reported that high biomethane productivity was achieved under nitrogen-limited biomass conditions with 462 ± 9 mLN CH_4_ g^−1^VS day^−1^ and the biomass-to-biomethane energy conversion efficiency was up to 84%.

Similarly, Yoo et al. [75] reported that osmotic shock enhanced the lipid recovery by two-fold in wet biomass of *Chlamydomonas reinhardtii*. In this technique, microalgae biomass was mixed with hexane, chloroform and methanol solvent in the Teflon-sealed tubes for 10 min for better lipid recovery since hexane and methanol required low heat for evaporation and rupture of microalgae cells [75]. It concluded that an osmotic shock is a promising technique for microalgae lipid extraction recovery from wet biomass.

### 4.2. Fatty Acids Extraction Method

Fatty acids (FA) are carboxylic acids with an aliphatic chain consisting of 4–26 carbons, joined by either saturated or unsaturated bonds [68]. There are several techniques to extract fatty acids from the microalgae biomass and convert it into fatty acid methyl ester (FAME) using gas chromatography-mass spectrometry (GC-MS), followed by subjecting it to a direct transesterification method; also, there is the Microbial identification (MIDI) system, Newark, Delaware, USA, the gravimetric method and the modified one-step in situ transesterification method (IST).

**Figure 6:**
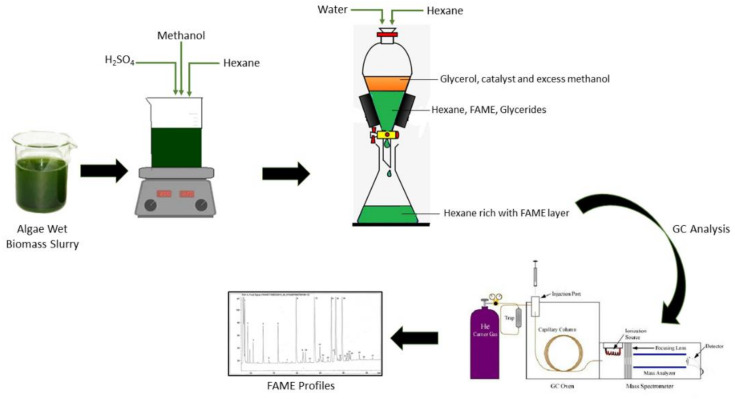
Microalgae fatty acids in situ transesterification.

A modified one-step in situ transesterification method (IST) was used to quantify fatty acid methyl ester (FAME) and it has been used by Zarrinmehr et al. [7] and Tang et al. [84]. Transesterification is the most common, less energy-consuming process to exchange pre-existent ester linkage using an acid or basic catalyst to release methanol and glycerol from synthesized fatty acid methyl esters [24]. According to Tang et al. [84], transesterification was related to the direct conversion of microalgal lipids into fatty acid methyl esters (FAME), either using acid or alkaline catalysts. This IST can be performed without lipid extraction technique (Figure 6). Besides that, this IST technique was widely used due to the high accuracy, simplicity and reliability. Generally, saturated fatty acids (SFA) possess a high cetane number and better oxidative stability. Microalgae with a high percentage of C18:1 were valuable components for biofuel production [85]. Higher cetane number is a prerequisite for reduced NO_2_ emission to the atmosphere [86]. According to Praveenkumar et al. [86], *Chlorella* sp. had potential as an alternative to biodiesel when cultivated in nitrogen-deficient conditions compared to phosphorus-limited conditions. An increase in unsaturated fatty acids is usually undesirable for biofuel production as it leads to a low cetane number, thereby enhancing NO_2_ emission, oxidation and lubricity of biofuels [86]. Hence, the biofuels must have a high amount of saturated and monounsaturated fatty acids with low levels of polyunsaturated fatty acids.

*Nannochloropsis gaditana* contained a high amount of eicosapentaenoic acid (EPA), followed by docosahexaenoic acid (DHA) content [84]. The *c* FAME yield of *N. gaditana* was 10.04% ± 0.08% by weight, with the EPA yields as high as 4.02% ± 0.17% by dry weight, which is approximately two times higher than the conventional method.

Besides that, another technique that is commonly used to quantify FAME is gas chromatography-mass spectrometry (GC-MS). Microalgae biomass was extracted first using solvents, and an aliquot of extraction was injected into the GC-MS capillary column at a specific time and pressure for the determination of fatty acid profile. Prommuak et al. [87] reported the fatty acids profile in *C. vulgaris* using GC-MS technique wherein the highest FAME obtained were methyl linoleate, methyl palmitate, methyl oleate and methyl stearate. Similarly, the study recorded that under nitrogen deprivation conditions, *Chlorella* sp. showed high amounts of saturated and monounsaturated fatty acids containing 36.45% stearic acid, 16.78% arachidic acid, 7.02% heneicosanoic acid and 19.91% linoleic acid. This study concludes that nitrogen starvation is suitable for the synthesis of high quality fatty acids for sustainable biodiesel production [86].

Cointet et al. [38] reported that FAME from *Entomoneis paludosa, Nitzschia alexandrina and Staurosira* sp. directly undergo transesterification using hydrochloric methanol and chloroform as solvent. The studies reported that the most abundant saturated fatty acid (SFA) was palmitic acid (C16:0), ranging from 32.5 ± 4.3% to 64.6 ± 6.0% for all strains in nitrogen-limited treatment. Whereas, the highest unsaturated fatty acid was oleic acid for *E. paludosa* (20.1 ± 3.2%) and palmitoleic acid for *N. alexandrina* and *Staurosira* sp., with 25.2 ± 1.9% and 25.0 ± 9.0%, respectively [38].

**Figure 7:**
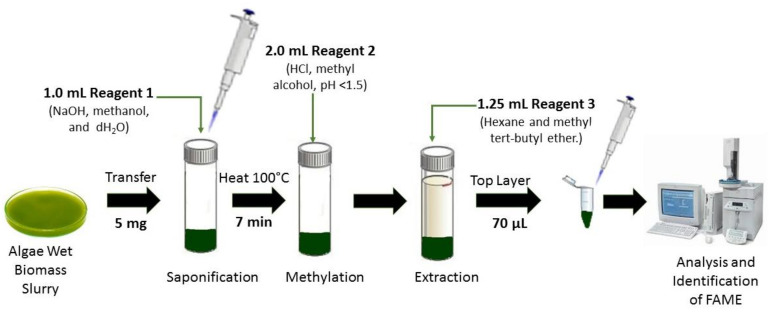
Microalgae fatty acids by Microbial Identification (MIDI) system.

Finally, FAME also can be analyzed using the MIDI system (Microbial ID, Newark, Delaware) as reported by Mulbry et al. [52]. They found that fatty acid content in algal turf scrubber (ATS) consisted of C14:0, 16:0, 16:1.7, 16:1.9, 18:0, 18:1.9, 18:2.6 and 18:3.3 when analyzed by the MIDI system. Figure 7 shows microalgae FAME analysis using the MIDI microbial ID technique.

The MIDI microbial identification system is a fully automated gas chromatographic analytical system, which identifies microalgae strains and bacteria based on their unique 9 to 20 carbons in length fatty acid profiles. Four reagents are required to cleave the fatty acids from lipids that involve several phases such as the saponification phase (sodium hydroxide, methanol and distilled water), methylation phase (hydrochloric acid and methyl alcohol), extraction phase (hexane and methyl tert-butyl ether) and sample cleanup phase (sodium hydroxide solution) before samples are injected to the GC port liner for analysis. The MIDI-FAME profile is an effective, rapid technique, and does not require concentrated biomass to be transferred from vial to vial [52,88,89].

## 5. Conclusions

This review has attempted to give an insight of the roles of nitrogen and phosphorus in microalgae cultivation for growth, biomass production, lipid yields and fatty acids. It gives a clear idea to the reader on how nutrients play a vital role in microalgae growth and their effects on biochemical composition. Microalgae exhibit higher growth rates and biomass production which might be an alternative source for bioproducts due to their high levels of lipids and fatty acids content in the cells. Besides that, cultivation media containing a high amount of nitrogen and phosphorus are more suitable for microalgae growth rates and for biomass production. Nutrient-limited conditions are more efficient for lipid and fatty acids generation. Other than that, suitable extraction techniques shall be implemented to achieve optimum lipid and fatty acids yield, reduce cost and have better bioproduct quality. All future research shall look into the optimization of nutrients in microalgae cultivation so that there will be more bioproduct generation and it can be commercialized for better usage in food, feed, fertilizer, nutraceutical and pharmaceutical applications.

## Figures and Tables

**Table 1:** Microalgae cultivation correlation with nitrogen content.

Microalgae Types	Experiment	Outcomes Growth and Biochemical Composition	Reference
*Nannochloropsis* sp.	Transferred from nitrogen-sufficient to -deficient condition	Lipid content increased from 32% to 60%	[27]
*Chlorella vulgaris*	Lipid production under nitrogen starvation conditions	Lipid productivity was 58.39 mg/L/day.	[28]
*Chlorella zofingiensis*	10 days cultivation in two conditions: nitrogen starvation and nitrogen repletion (0 g/L and 1.1 g/L, respectively)	Rapid growth in nitrogen-sufficient culture medium, whereas growth inhibition under nitrogen starvation condition	[3]
*Scenedesmus* sp.	Effects of nitrogen limitation and starvation on morphological and biochemical changes	Nitrate starvation generates 27% lipid accumulation	[29]
*Nannochloropsis oceanica*	14 days under nitrogen-deplete and nitrogen-replete conditions	Regulation of metabolic pathways along with acetyl CoA synthesis and lipid degradation for TAG synthesis	[30]
*Chlorella* sp. and *Nannochloropsis oculata*	13 days in Conway nutrient batch cultures without nitrogen in the 7th day of growth	15.3 ±1.0% lipid in *Chlorella* sp., 33.7 ± 2.8% lipid in *N. oculata* under nitrogen limitation	[31]
*Phaeodactylum tricornutum*	Lipid accumulation in nitrogen-deficient medium	High lipid content (53.04 ± 3.26%)	[5]
*C. reinhardtii*	Effect of different nutrients on standard TAP, TAP nitrogen deficiency (T-N), TAP without nitrogen and phosphorus (T-N-P), TAP nitrogen deficiency with additional phosphorus 1 M(K)PO_4_ (T-N+P) on growth and lipid accumulation	Lipid contents in cells increased as a result of nitrogen-deficient conditions; the highest lipid content was 104.7% in T-N-P condition and the lowest lipid values were 49.9% in T-N condition	[32]
*I. galbana*	Effect of different nitrogen concentrations(0, 36, 72, 144 and 288 mg/L) on the growth rate and biochemical composition	Growth decreased in nitrogen-deficient conditions; in contrast, carbohydrates and fatty acids showed the highest value, 47% and 75%, respectively	[7]
*Scenedesmus obliquus*	Various nitrogen concentrations’ effect on growth and lipid production	Lipid production increased with increasing nitrogen concentration; the highest cell density (1.7 × 10^7^ cells/mL) and lipid production (242.4 mg/L)	[33]

**Table 2:** Effect of nitrogen availability and starvation conditions on biomass yield, lipid content and fatty acids in algae.

Microalgae Strain	Initial Nitrogen Available	Final Nitrogen Starvation	Cultivation of Algae (Days)	Biomass Yield	Lipid Content	Fatty Acid	Higher Fatty Acids	Reference
*S. obliquus*	0 mM	--	14	719 mg/L/d	35%	44.7 ± 1.7%	C18:1	[35]
*C. zofingiensis*	0 mg/L	--	10	0.7 g/L	24.5%	12.10 × 10^−7^ µg 10.49 × 10^−7^ µg 6.25 × 10^−7^ µg	C16:0, C18:1, C18:2	[3]
*Scenedesmus quadricauda*	0 mg/L	--	30	265 × 10^4^ cells/mL	226 mg/L	15.16%, 25.08%, 15.78%	C18:0, C18:3 C20:0	[36]
*Ankistrodesmus falcatus*	750 mg/L	--	14	1740 mg/L	59.6%	24.68 ± 0.13%, 19.2 ± 0.66%, 16.64 ± 0.33%, 11.77 ± 0.73%	C16:0, C18:1, C18:2, C18:3	[37]
*Phaeodactylum tricornutum*	0 mg/L	--	21	68.57 ± 7.57 mg/L	53.04 ± 3.26%	17.73 ± 8.40%, 13.07 ± 6.03%, 1.81 ± 1.25%	C16:0, C16:1, C20:5	[5]
*Entomoneis paludosa*	83.56 μM	0.14 μM	35	2.00 ± 0.11 cells/mL	20.68 ± 2.62%	36.7 ± 6.9%, 13.5 ± 2.0%, 20.1 ± 3.2%	C16:0, C18:0, C18:1	[38]
*Rhodomonas* sp.	1.5 g/L	113 mg/L	8	2.5 × 10^6^ cells/mL	30.3%	6.1%, 5.5%, 3.7%	C16:0, C18:1, C18:3	[39]
*Nannochloropsis oceanica*	0 g/L	NR	3	20 × 10^6^ cells/mL	58%	48%, 40%, 5%	C16:0, C16:1, C18:1	[8]
*Picocystis salinarum*	9.375 mg/L	--	20	0.7 g/L	33.87%	72.13 µg/g, 923.95 µg/g, 19.87 µg/g	C14:0, C16:0, C18:2	[40]

**Table 3:** Microalgae cultivation in relation to phosphorus content.

Microalgae Types	Experiment	Outcomes	Reference
*Eustigmatophyte sp.*	Phosphorus limitation condition	Triacylglycerols, TAG content increased from 12.9% to 15.1% in absence of phosphorus condition	[46]
*C. zofingiensis*	Affects phosphate on lipid accumulation as a biodiesel feedstock	Lipid content obtained from phosphate-deficient was 44.7%, relatively higher than cells grown in full medium (33.5%).	[34]
*C. vulgaris*	Biodiesel production under phosphorus-sufficient conditions	Lipid yield of *C. vulgaris* for biodiesel production was 58.39 mg/L/day in phosphate-sufficient conditions	[28]
*I. galbana*	Varied levels of phosphorus in F/2 medium for biodiesel production	Phosphorus starvation stimulated lipid accumulation up to 50% *w*/*w*	[47]
*Scenedesmus* sp.	Phosphorus-rich medium from public market wastewater treated for 8 days	Total phosphorus in medium reduced about 82.17%	[48]
*Chlorella ellipsoidea* and *Chlorococcum infusionum*	Phosphorous starvation culture for 30 days	Total lipid content of *Chlorella* (41.8 ± 1.9% at 16 days of incubation period) and *Chlorococcum* (31.3 ± 1.0% at 18 days of incubation period)	[49]
*Scenedesmus obtusiusculus*	Cultivated in BG-11 medium under non-limiting conditions	6.4% *w*/*w* lipid yield after SCCO_2_ extraction	[50]
*Scenedesmus* sp.	Resuspension in modified SE medium with 2 mg/L NaH_2_PO_4_·_2_H_2_O for 9 days	The highest lipid production (350 mg/L) and lipid content (41.0%) were obtained by addition of 2 mg/L NaH_2_PO_4_·_2_H_2_O every 2 days	[6]
*Chlorella* sp.	Lipid enhancement through nutrient starvation	Under phosphate starvation, the lipid content was 13.9%.	[51]

**Table 4:** Effect of phosphorus availability and starvation conditions on biomass yield, lipid content and fatty acids in algae.

Microalgae Strain	Initial Phosphorus Available	Final Phosphorus Starvation	Cultivation of Algae (Days)	Biomass Yield	Lipid Content	Fatty Acid	Higher Fatty Acids	Ref
*Phormidium* sp.	0.02 g/L	--	15	0.130 g	27.9%	34.667%, 11.266%, 27.912%,	C16:0, C18:0 C20:2,	[54]
*Chlorella* sp.	32 μM	--	22	2 g/L	23.60%	35.48%, 33.90%	C16:0, C18:0	[55]
*C. ellipsoidea, C. infusionum*	1.5 g/L	--	30	1.56 ± 0.06 g/L, 2.17 ± 0.12 g/L	41.8 ± 1.9%, 31.3 ± 1.0%	21.62 ± 0.94%, 30.32 ± 2.68%	C18:1,	[49]
*Rhodosporidium kratochvilovae*	0.05 g/L	--	10	12.65 ± 0.12 g/L	51.7 ± 0.81%	10.36 ± 0.59% 66.79 ± 0.21% 10.48 ± 0.43%	C18:0, C18:1, C18:2	[32]
*C. reinhardtii*	0 μg/mg	--	7	1.1 g/L	105.00 μg/mg	35.86 ± 0.25 μg/mg, 22.12 ± 0.12 μg/mg, 22.84 ± 0.08 μg/mg	C16:0, C18:2, C18:3	[4]
*Scenedesmus* sp.	2mg/L	--	9	0.6 g/L	41.0%	NR	C16:0, C18:1	[6]
*C. reinhardtii*	0.4 mg/L	--	14	0.08 mg/L	NR	56% 28.8%	C16:0, C18:3	[56]
*Nannochloropsis oceanica*	0 g/L	--	6	2.5 mg/L	337 mg/g	37.3 ± 0.1%, 30.8 ± 0.3%, 10.7 ± 0.8%.	C16:0, C16:1, C20:5	[57]
*N. oceanica*	0 g/L	--	14	0.29 g/L	23.7%	13.93%, 26.31%	C18:1, C16:1.	[41]

**Table 5:** Techniques used to quantify lipids in microalgae [64].

Types of Extraction Method	Advantages/Efficiency	Key Methodology	Drawbacks
**Mechanical Approach**			
Expeller press	Simple and effective crushing method	High mechanical pressure to crush the cells and squeeze oil out from microalgae biomass	Pressure decreased lipid recovery, increased heat; expensive and time-consuming
Ultrasound-assisted extraction	Economical and eco-friendly, completed in a short time, high reproducibility, does not require the addition of beads or chemicals	Cavitation produces heat shock waves to disrupt microalgae cells with less thermal denaturation of biomolecules	Expensive, leads to free radicals and detrimental to the oil quality
Bead beating	Moderate efficiency	Disruption of cells using high-speed spinning beads	Moderate efficiency
Microwaves irradiation method	Short reaction time, low costs, efficient extraction and quick oil recovery	Extraction and transesterification of the oils into biodiesel by intracellular heating, which disrupts the cells	High maintenance cost on a large scale.
Electroporation	High efficiency	Altered cell membranes and cell walls to improved lipid extraction	High initial and maintenance costs
**Chemical Approach**			
Supercritical fluid extraction method (SEF)	Rapid, safe and economical method and does not require dewatering of biomass	Using high pressure to extract lipids from cells	High cost and environmental and safety issues
Bligh and Dyer method	Rapid and easy processing of large number of samples	Extracting total lipids from microalgae using ratio 2:1 methanol: chloroform	Less sensitive
Pressurized solvent extraction	High efficiency	Using solvent and pressurized nitrogen for extraction	High cost, fire, health and environmental hazards
Folch method	Rapid and easy processing of large number of samples	Combination of different solvents such as chloroform–methanol in the ratio of 2:1 by volume	Less sensitive
Soxhlet extraction method	Easy processing of a large number of samples and moderate efficiency	Normally used to quantify high quality lipids such as fatty acids and triglycerides using hexane as a solvent	Environmental and health risks
Accelerated solvent extraction (ASE)	Short process time and can recover solvent for reuse	Using heat or pressure to achieve better lipid recovery	Less efficient in a larger scale
